# Occurrence of claw asymmetries in fattening pigs and potential impact on the development of sole ulcerations

**DOI:** 10.1186/s40813-022-00281-y

**Published:** 2022-08-29

**Authors:** Sarah Seufert, Nina Volkmann, Johannes Schmidt-Mosig, Nicole Kemper

**Affiliations:** 1 Institute for Animal Hygiene, Animal Welfare and Farm Animal Behavior, University of Veterinary Medicine Hannover, Foundation, Hannover, Germany; 2 Science and Innovation for Sustainable Poultry Production (WING), University of Veterinary Medicine Hannover, Foundation, Vechta, Germany; 3 vetvise GbR, Rastede, Germany

**Keywords:** Claw asymmetries, Claw sole ulceration, Claw health, Pigs, Animal welfare

## Abstract

**Background:**

Claw abnormalities, particularly claw asymmetries, are associated with lameness in pigs and can be a welfare issue. However, the prevalence and development of claw asymmetries in pigs of different age is unknown. Therefore, the aim of this study was to analyze the claw symmetry over the pig lifetime from birth to slaughter as well as the occurrence of sole ulcerations in fattening pigs possibly caused by such asymmetric claws.

**Results:**

From third day of life until slaughtering, asymmetric growth of the claws was detected more frequently and more severely with increasing age as determined by three-step scoring. Sole ulcerations were detected in slaughtered pigs only with a prevalence of 64.2% (197/307 examined animals). The risk for a sole ulceration was 3.6-fold higher for pigs with strongly asymmetric claws (≥ 30% size difference of the claw footing area) compared to slightly asymmetric claws (≥ 5–15% size difference of the claw footing area) (odds ratio (OR) = 3.6). It was even higher for pigs showing intermediately asymmetric claws (≥ 15–30% size difference of the claw footing area) (OR = 2.7).

**Conclusions:**

The study showed a significant increase in the prevalence of claw asymmetries over the pigs’ lifetime, which can lead to serious pathologic findings with increasing age such as sole ulcerations. Most likely, the unbalanced weight load on single claws in combination with hard flooring can result in claw damages. Moreover, a genetic component cannot be excluded because claw asymmetries were already detected in suckling piglets.

## Background

Lameness is an important welfare issue in livestock farming because it is associated with pain and suffering of the affected animals [1–4]. According to Whay et al. [4], lameness is the most important indicator for animal welfare assessment in pig farming. In addition, lameness and the resulting impact on animal welfare has an economic impact as reflected in the reduced production capacity and decreased longevity of sows [2, 5–8], worse feed conversion [9], or early culling of the animals [3, 10].

Frequent claw lesions can lead to lameness in pig husbandry [1, 3, 10]. Many claw pathologies such as sole bruising, sole hemorrhages, erosions, wall cracks, white line defects, sole ulceration, and claw joint inflammation exist [11, 12]. These can all lead to varying impacts on animal welfare by pain and functional impairments depending on their severity [3]. The development of claw lesions is generally multifactorial [13, 14]. Claw lesions have a genetic component in addition to external influences such as housing conditions especially floor conditions [15], nutrition [7], as well as management and infectious diseases [13, 16].

Uneven and asymmetric claws are characterized by a disproportion between digit and metacarpus III and IV of the pig’s claw, e.g., with longer digits and metacarpus IV. This results in a wider sole [16]. Such claw asymmetry in pigs was first described by Nordby [17] who pointed out that with unevenly sized claws, a greater weight load is placed on the outer claw due to unequal weight distribution. This leads to a subsequent incorrect load on the joints of the entire limb [17]. In most cases, lateral claws were found to be longer than the medial ones [17–19]. Furthermore, asymmetric claws are predisposed to injuries and lesions [16, 17, 19–21]. In a study of pathomorphological and radiological examinations of claws of 1702 slaughter pigs, asymmetric claws were found in 46.37% of the examined animals [16]. The asymmetry occurred significantly more frequently and seriously in the claws of the hind limbs than the front limbs [16, 22]. A difference in size was documented between the inner and outer claw, but this was only seen in the claws of the hind limbs; these differences were not detected in the inner and outer forelimb claws [22]. Most probably, the larger outer claw of the hind limbs carries more weight than the inner claw, and is thus subjected to more mechanical stress [22]. Moreover, it is assumed that claws’ asymmetry partially has a genetic origin [17, 18].

A study on Czech and Belgian Landrace pigs showed that differences in size between lateral and medial claw were related to the breed; a recessive inheritance was suspected [16]. Studies on wild boar claws showed differences in size between the claws of the fore and hind limbs, but no asymmetries were found in the lateral and medial claws [23]. Genetic selection in domestic pig breeds was assumed to be the origin of these anomalies in light of the lack of claw asymmetries in wild boars [23]. Furthermore, claw asymmetries were found in both cattle and pigs kept on hard floors, thus showing a higher percentage than wild boars or cattle kept on soft floors [19, 22, 24]. The literature suggests that the predisposition to asymmetric claws is hereditary, but the actual occurrence is promoted by housing the animals on hard floors. The combination of unbalanced and asymmetric claws with uneven weight loading on the claws and hard flooring in intensive livestock farming can lead to other claw pathologies such as sole ulcerations [16, 17, 19–21]. In turn, severe sole ulcerations lead to pain and lameness of the animals, which impair animal welfare [25, 26].

The aim of this study was to record the occurrence of claw asymmetries in fattening pigs especially how these asymmetries develop over the pigs’ lifetime. Furthermore, the occurrence of sole ulcerations in these fattening pigs was analyzed in relation to asymmetric claws. Thus, more information on the actual occurrence of asymmetric claws in German pig herds should be gained.

## Results

### Claw asymmetries

The calculation of observer reliability for the scoring of asymmetries resulted in a Krippendorffs’ alpha of α = 0.93 (CI 0.86–0.98), which represents a very good agreement.

**Fig. 1:**
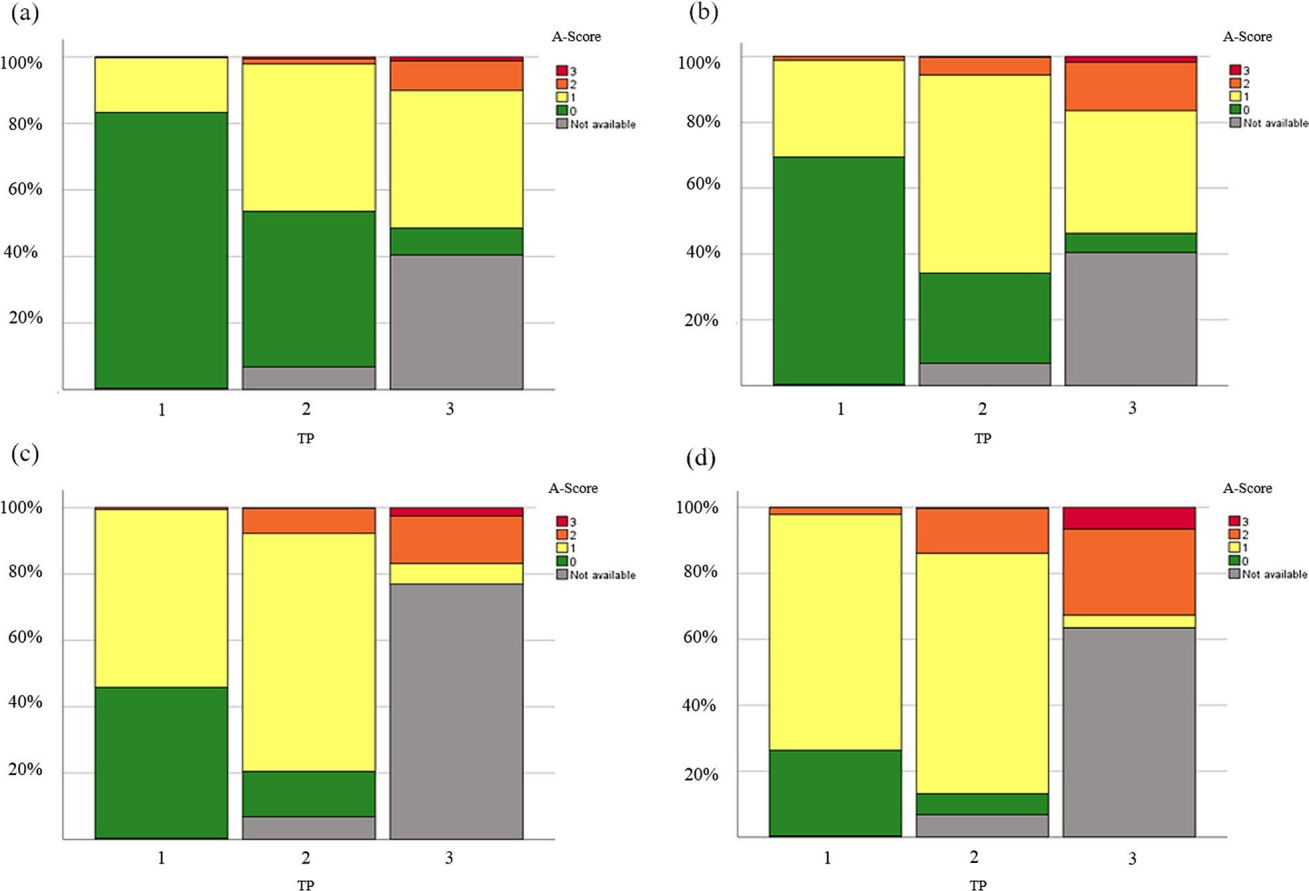
Prevalence of claw asymmetries at the three observation time points. Prevalence of asymmetries at each claw (**a** = left anterior, **b** = right anterior, **c** = left hind, **d** = right hind) at the three different time points (TP) (1 = 3rd day of life, 2 = 4th week of age, 3 = at slaughter) based on the total number of pigs initially included in this study. Claw numbers at the different TP were: TP1: n = 515; TP2: n = 482; TP3: **a**, **b** n = 307; **c** n = 118; **d** n = 189). The A-Scores were given according to a system with a Score 0 representing no asymmetry, Score 1 showing claws with slight asymmetries, Score 2 with intermediate asymmetry, and Score 3 with strong asymmetry as well as the indication “not available” classifying animals that died or were sold as well as the one hind claw, which was not photographed at the slaughterhouse due to technical limitations. The above n-numbers are the available claw numbers minus the claws of the animals that were not available

The frequency of the A-Scores 0–3 at three different observation times is shown in Fig. 1a–d for each of the four claws based on the total number of pigs initially included in this study. Although several animals could no longer be examined at the third observation time—and only one of the hind claws was considered in the evaluation—these results suggest an increase in claw asymmetries over time.

The results regarding the total number of evaluated pigs are shown in Table 1. At the first observation time point, most of the anterior claws showed no asymmetry (A-Score 0) (prevalence left: 83.3%, right: 69.3%); the majority of the hind claws were scored as slightly asymmetric (A-Score 1) (prevalence left: 53.8%, right: 71.9%). At this point, no strongly asymmetric claws were detected.

**Table 1:** Prevalence (%) of pig claw asymmetries classified using the A-Score scoring system

A-Score (%)	Time point 1 (n = 515)				Time point 2 (n = 482)				Time point 3 (n = 307)			
	0	1	2	3	0	1	2	3	0	1	2	3
LAC	83.3	16.5	0.2	0.0	50.2	47.5	1.7	0.6	13.6	69.5	14.9	2.0
RAC	69.3	29.5	1.2	0.0	29.5	64.5	5.8	0.2	9.7	62.7	24.7	2.9
LHC	45.6	53.8	0.6	0.0	14.7	77.0	8.1	0.2	0.0	26.9	62.2	10.9
RHC	26.0	71.9	2.1	0.0	6.9	78.2	14.5	0.4	0.0	10.7	71.3	18.0

A-Score 0 = no asymmetry, A-Score 1 = slight asymmetry; A-Score 2 = intermediate asymmetry; A-Score 3 = strong asymmetry; time point 1 = 3rd day of life; time point 2 = 4th week of age, time point 3 = at slaughter). Values (%) are given for each individual claw (LAC left anterior, *RAC* right anterior, *LHC* left hind, and RHC = right hind) in relation to the total number of evaluated pigs

At 4 weeks of age, the number of slightly and intermediate asymmetries (A-Scores 1 and 2) increased in all four claws showing that hind claws were more frequently affected than anterior ones. The first strongly asymmetric claws (A-Score 3) were detected on the front legs (prevalence left: 0.6%, right: 0.2%) at this time.

In slaughter animals, only a small percentage of the front claws remained symmetric (A-Score 0) (prevalence left: 13.6%, right: 9.7%); the majority showed a higher degree of asymmetry (A-Score 2 and 3) or were slightly asymmetric (A-Score 1). All hind claws of the slaughtered animals were asymmetric—most had an intermediate asymmetry (A-Score 2) (prevalence left: 62.2%, right: 71.3%). Strongly asymmetric claws (A-Score 3) were found in the hind claws of the slaughtered pigs in up to 18.0% (right hind claws) (prevalence left hind claws: 10.9%). The prevalence of asymmetries in the hind claws increased versus anterior ones over the course of the study period. The right claws showed more frequent and stronger asymmetries than the left ones for both anterior and hind claws.

### Sole ulcerations

The calculation of observer reliability for the scoring of ulcerations resulted in a Krippendorffs’ alpha of α = 0.87 (CI 0.75–0.97) suggesting very good agreement. Sole ulcerations were detected at the third observation point in slaughtered pigs. Suckling piglets and animals aged 4 weeks did not show any sole ulcerations. The prevalence of sole ulcerations in slaughtered pigs was 64.2%. The results for the prevalence of sole ulcerations are listed in Table 2.

**Table 2:** Prevalence (%) of sole ulcerations in pigs classified using the U-Score score system

U-Score (%)	0	1	2	3
LAC (n = 307)	60.6	55.4	65.2	56.1
RAC (n = 307)	25.1	27.7	21.2	30.1
LHC (n = 118)	11.7	13.3	11.9	12.2
RHC (n = 189)	2.6	3.6	1.7	1.6

U-Score 0 = no visible sole ulceration; U-Score 1 = slight sole ulceration; U-Score 2 = intermediate sole ulceration; U-Score 3 = strong/severe sole ulceration. Values (%) are given for each individual claw (*LAC* left anterior, *RAC* right anterior, *LHC* left hind, *RHC* right hind) in relation to the total number of evaluated claws

The results of the calculated odd ratios (OR) for the occurrence of sole ulcerations (U-Class 1) in pigs with detected claw asymmetries are shown in Table 3. Pigs with strongly asymmetric claws (A-Score 3) showed a significant (3.6-fold) increased risk (OR = 3.6) for at least an intermediate sole ulceration (≥ U-Score 2) versus those with slightly asymmetric claws (A-Score 1) (Table 3). Furthermore, the risk for sole ulcerations was 2.7-fold higher (OR = 2.7) in pigs having strong asymmetric claws (A-Score 3) than in animals with intermediate asymmetric ones (A-Score 2).

**Table 3:** Relation of claw asymmetries and claws with U-Class 1

	Odds Ratio	Confidence limits		*P*-value
A-Score 1 versus A-Score 2	1.307	0.588	2.907	0.5116
A-Score 1 versus A-Score 3	3.553	1.428	8.843	0.0064
A-Score 2 versus A-Score 3	2.719	1.472	5.023	0.0014

A-Score 1 = slight asymmetry; A-Score 2 = intermediate asymmetry; A-Score 3 = strong asymmetry; U-Class 1 representing those claws with intermediate or strong sole ulceration (previously U-Score 2 and 3)

## Discussion

There are relatively few existing studies on claw asymmetries in pig, and most of them are based on mother sows and gilts [22] or single claws from slaughtered animals [16, 20, 27]. This study investigated the occurrence of claw asymmetries during the lifetime of fattening pigs at three different observation time points: the first days of life, at 4 weeks of age, and after slaughtering. Observing the claws of suckling piglets answers the question of whether claw asymmetries already occurred in the first days of life, and which degree of asymmetry already existed shortly after birth. To assess further development of asymmetries, pigs’ claws were also evaluated at the time of weaning and at slaughter.

This work scored the claws using photographs. This procedure enabled the observer to score the claws as accurately as possible while minimizing the stress to the animals by fixing them only briefly. Likewise, data collection via photographs was successfully used in previous clinical studies [28, 29]. Blömke et al. [29] evaluated ear and tail lesions on pictures of fattening pigs taken at the abattoir. To describe the inflammatory and necrotic manifestations in new-born piglets, Kuehling et al. [28] recorded the animals with a camera and performed clinical scoring based on those photographs. Upon scoring claw lesions in sows, van Riet et al. [12] stated that using digital recordings for scoring was faster during collection because an observer could score the recordings immediately afterwards. However, the observer could not touch or manipulate the claws and, therefore, some lesion types were less visible [12]. But, scoring using photographs has the advantage that different observers can evaluate the same animal/claw without having to be in the same place at the same time. Furthermore, such photographs can also be used to test inter-observer agreement as performed here. The high agreement in observer reliability confirmed the utility of our scoring method.

Using photographs to score claws also allowed us to compare the development of asymmetries over the animal’s lifespan. For reasons of animal welfare and production procedures, the pictures of the claws of slaughtered pigs could only be taken at the slaughter line after pricking and before scalding. Consequently, all slaughtered animals were fixed by the right or left hind limb, which could thus not be photographed. To show the development of prevalence and severity over time (Fig. 1), the asymmetries at all three observation points were calculated while referring to the same number of animals as originally included in this study (n = 517). The animals eliminated from the study at the two later time points were indicated as “not available”. Another possibility would have been to evaluate only the data on pigs observed up to the point of slaughter. However, we wanted to include as many animals as possible in the study and decided to use the method presented here. In doing so, we examined the claws of over 500 piglets and showed that claw asymmetries were already present in suckling piglets. They became more frequent and severe with increasing growth and thus age.

The fact that new-born piglets showed claw asymmetries with a partially intermediate grade of asymmetry indicated a genetic component. This is consistent with previous studies [16, 18]. Many claw pathologies in pigs are multifactorial [13, 14, 20, 30], e.g., by flooring [14, 15, 31], husbandry conditions such as regrouping of the animals [30, 32], climate and hygiene [33], or nutrition [7]. Therefore, the development of claw asymmetries should also be examined from several points of view. For example, Grandhi et al. [27] studied the influence of nutritional factors and structural unsoundness on the inequality of the digits of the claws of front and hind limbs in swine. They concluded that nutrition had only minor impacts, and structural unsoundness had no influence on the different lengths of the limb digits [27]. Here, the claws of the right limb were affected more frequently and more severely by asymmetry than the left ones; this was seen in both anterior and hind limbs. This finding was detected at all three observation times. This observation has not been described before, but was seen even in new-born suckling piglets, which also indicates a likely genetic component. However, we cannot provide a sound explanation for this result.

Similar to previous studies [16, 20, 22], our results showed that the claws of the hind limbs were more frequently asymmetric than the anterior ones. However, size differences between the inner and outer claws of a limb were also observed on the forelimbs, which contradicted the results of Van Amstel and Doherty [22]. We note that Van Amstel and Doherty [22] examined the claws of only three single gilts (113–150 kg). Here, slaughter pigs with symmetric front claws were also found. The right and left hind claws of all slaughter pigs were all asymmetrical without exception. Furthermore, these had the highest percentage of strong/severe asymmetry.

Previously de Carvalho et al. [34] measured pig claw pressure distribution and found a significant unequal distribution of pressure between the two claws of the hind limbs with a higher load on the outer claw. This might also explain the strong asymmetries in claws of slaughter pigs seen here. The unequal weight distribution—with single claws differing in size from each other—may have resulted in an automatically different load on the individual pair of claws. The overloading of the individual claws may have predisposed the development of other claw pathologies such sole ulceration. This assumption was already made by Mouttotou et al. [35] who examined 3,974 slaughter pigs for claw pathologies and determined that hind claws were most frequently affected by claw lesions. Other previous studies indicated that claws differing in size and lead to unequal weight distribution and tend to favor the development of claw pathologies [19, 21].

Here, sole ulcerations were counted as all visible erosions of the claw sole. These include low-grade sole defects with dark red pigmentation due to haemorrhage and abrasion of the sole surface up to profound loss of substance and necrosis of the sole tissue. Sole ulcerations were detected in slaughter pig while suckling piglets and 4-week-old animals were not affected. Thus, we assumed that the size and weight of the animals impacted claw pathology. Lippuner [20] investigated claw diseases in Swiss fattening pigs, boars, and sows, and found more severe claw lesions in larger and heavier pigs. The prevalence of sole ulcerations in slaughtered pigs seen here was 64.2%. However, only a visual assessment of sole ulcerations was carried out in this study. Therefore, the impact of these findings on animal welfare could not be evaluated because pathohistological diagnoses were not assessed. However, it is known that even superficially visible claw lesions can cause pain and discomfort in animals although some claw lesions are only visible on pathohistological examination [36].

**Fig. 2:**
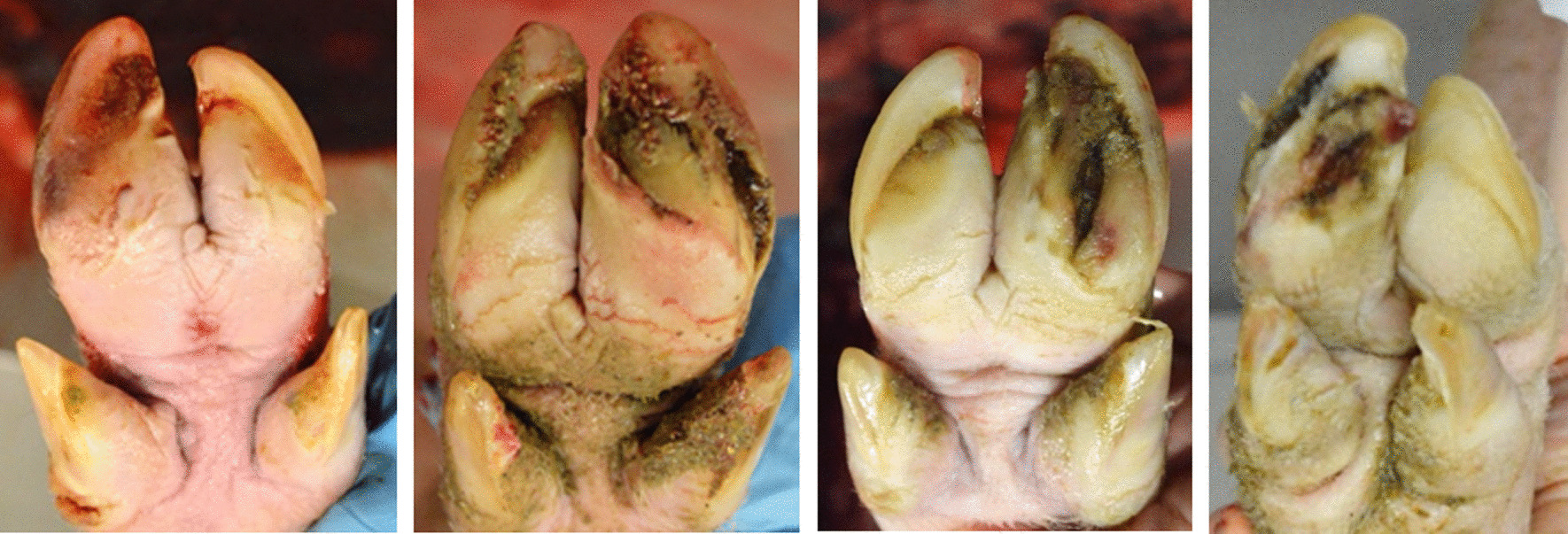
Representative photographs of claws from slaughter pigs with strong asymmetries (A-Score 3) and U-Class 1. U-Class 1 = moderate to severe sole ulcerations of the larger main claw; pictures from left to right: right front claw, left front claw, left front claw, right hind claw) (pictures taken by Sarah Seufert)

In cattle, sole ulcerations are among the most common causes of lameness; the extent of lameness depends on how deep the ulcers reach in the tissue layers of the soles [37]. Stracke et al. [38] investigated the relation of visible lesions on turkey’s foot-pad and the histopathological parameters. They showed that the severity grade of ulcerations was significantly related to the size of the alteration (percentage in relation to the footpad). Analogous to Stracke et al. [38], one could conclude that the visible claw lesions may reflect the histopathological alterations subcutaneously as well. However, to confirm the visual ulceration score, a pathohistological analysis of the claw sole is necessary to determine the real extent of tissue damage. Nevertheless, ulcerations of the sole in grade 2–3, with clear visual pathological lesions (Fig. 2), were assumed to cause pain to the animal with a negative impact on animal welfare.

The results showed that animals with strong claw asymmetries had an increased risk of being affected by sole ulcerations. Thus, we concluded that asymmetric claws have a significantly higher risk of developing sole ulcerations, which in turn impacts animal welfare depending on their severity. Claw asymmetries in fattening pigs can lead to further pathologies causing pain, and thus this deformation should be prevented or at least reduced.

Lippuner documented a high to very high heritability for shape and size of the claws of fattening pigs of different breeds [20]. For fattening pigs already affected by claw asymmetries, the community should investigate to what extent prophylactic measures can be taken at rearing age to prevent future claw damage in fattening pigs. There are multifactorial causes leading to claw pathologies; thus it is not possible to solve this issue only by improving the husbandry conditions, e.g., floor type [14]. However, a softer surface flooring such as rubber mats could reduce possible secondary pathologies such as sole ulcerations [39].

Díaz et al. [40] showed that sows with overgrown claws had more frequent claw erosions than sows with claw maintenance/trimming. Therefore, similar to cattle [41], claw care/trimming is an important component of claw health and animal welfare—especially in animals with asymmetric claws. Feed supplements such as biotin and zinc can support claw health [42, 43] and can potentially reduce the development of claw lesions associated with asymmetric claws.

## Conclusions

This study showed a significant increase in the prevalence of claw asymmetries over pigs’ lifetime, which can lead to serious pathologic findings, such as sole ulcerations, over the animals’ lifespan. Overall, the claws of the hind limbs were more frequently and more severely affected by asymmetries than those of the forelimbs. The unbalanced weight load of the asymmetrical claws in combination with hard floors likely damaged the claws including the claw sole ulcerations seen in slaughter pigs. A genetic component must be considered because claw asymmetries were already detected in suckling piglets. Further studies are needed to precisely determine the development of claw asymmetries in pigs and find the appropriate prophylactic measures to prevent them such as husbandry optimization.

## Materials and methods

### Animals and study location

The study was conducted on a German research farm and its slaughterhouse from September 2019 to September 2020. The Boxberg Teaching and Research Centre (LSZ), a subunit of the Ministry of Rural Affairs and Consumer Protection of the Federal State of Baden-Württemberg in Germany, is a research facility for pig breeding, rearing, and fattening; the LSZ maintains different conventional and alternative housing systems for pigs. Here, only animals in conventional systems were observed.

A total of 517 pigs (Breed: German Landrace × German Edelschwein × Pietrain) were examined in five sequential batches. Each of the five groups consisted of piglets of litters of eight sows corresponding to 90–100 animals per group. The pigs were included in this study over their entire lifetime from suckling pigs to slaughter. Due to death, culling, and withdrawal of underweight animals during the rearing process, the data on 517 pigs remained with 96–107 pigs per group (Table 4).

**Table 4:** Animals in the five experimental groups involved in the study

	Time point 1							Time point 2							Time point 3						
Group	∑	n ♂	n ♀	LAC	RAC	LHC	RHC	∑	n ♂	n ♀	LAC	RAC	LHC	RHC	∑	n ♂	n ♀	LAC	RAC	LHC	RHC
1	104	48	56	104	104	104	104	93	40	53	93	93	93	93	67	27	40	67	67	32	35
2	96	39	57	96	96	96	96	94	37	57	94	94	94	94	60	19	41	60	60	13	47
3	104	57	47	104	104	104	104	96	43	53	96	96	96	96	66	31	35	66	66	26	40
4	104	54	50	104	104	104	104	98	48	50	98	98	98	98	60	27	33	60	60	28	32
5	107	46	61	107	107	107	107	101	42	59	101	101	101	101	54	22	32	55	55	20	34
∑	515	244	271	515	515	515	515	482	210	272	482	482	482	482	307	126	181	307	307	307	307

Time point 1 = 3rd day of life, time point 2 = four weeks of age, time point 3 = at slaughter; these numbers include the number of each individual claw. Numbers are given for the sum of the investigated animals (∑), grouped by sex (n ♂ = male; n ♀ = female) as well as of each individual claw (*LAC  * left anterior, *RAC* right anterior, *LHC* left hind, *RHC* right hind)

Sows were kept in a farrowing crate for farrowing and subsequent lactation. Sows within the group farrowed with approximately three days variation, i.e., non-induced group farrowing; this resulted in varying piglet ages per group. The overall pen size was 4.80 m^2^. The pen was equipped with a plastic-coated metal stretch floor, a piglet nest (0.86 m^2^) with zone heating, and a heat lamp. The suckling period lasted 28 days. Piglets were given milk replacer via feed troughs in large litters or during insufficient milk production by the sow. Piglets were fed pre-starter starting on the second week of life.

Two piglets were moved due to litter balance in one of the five sow groups. After the second week of life, small connecting doors were opened for the piglets between the farrowing crates of four sows. Thus, the piglets of the four sows each spent the remaining 2 weeks of the farrowing period together with access to all four mother sows.

Male piglets were castrated between the second and fourth day of life; the tails were not docked. The teeth were ground as needed. All piglets got an individual ear tag. On their third day of life, the piglets received a subcutaneous iron application to prevent iron deficiency anemia. On the 14th day of life, piglets were vaccinated against PRRS (Porcilis® PRRS, MSD Tiergesundheit, Intervet Deutschland GmbH, Unterschleißheim, Germany) per intradermal, needleless vaccination. On the 21st day of life, they received a vaccination against *Mycoplasma hyopneumonia* (Hyogen®, Ceva Tiergesundheit GmbH, Düsseldorf, Germany), porcine circovirus (Porcilis® Glässer, MSD Tiergesundheit, Intervet Deutschland GmbH, Unterschleißheim, Germany) and *Escherichia coli* (Entericolix®, Boehringer Ingelheim Vetmedica GmbH, Ingelheim am Rhein, Germany).

Body weights were recorded directly after birth, on the 21st day of life, and before weaning at the age of 4 weeks. The average body weight at weaning was 7.5 kg.

For piglet rearing, the animals were housed in 22 m^2^ pens with a combination of plastic and concrete slatted floor at a ratio of 50:50. There were 48 animals in each pen resulting in a space allowance of about 0.45 m^2^ per animal. Feeding and drinking was provided by liquid feed system and nipple drinkers.

The animals were vaccinated against *Actinobacillus pleuropneumonia* (Suivac® APP, Chem Vet dk A/S, A.C., Silkeborg, Denmark) at the age of 6 and 10 weeks. The rearing period lasted from 4 weeks to 11 weeks and the starting body weight was 7.8 kg on average with a body weight of 28 kg at the end of the rearing period. The piglets were transferred to the fattening pens after 11 weeks.

During their fattening period until an age of 21–28 weeks, all animals in the five batches were kept in 22 m^2^ pens with a concrete slatted floor. Eighteen pigs were housed with a space allowance of 1.19 m^2^ per animal. Feeding and drinking was provided by liquid feed systems and nipple drinkers.

All fattening animals were weighed when they were moved into the fattening pen. In addition, an intermediate weighing took place at 19 weeks to define the approximate slaughter date. When reaching a final fattening weight of 113–120 kg, they were slaughtered at the LSZ abattoir where slaughtering occurs once a week. The animals were anesthetized with carbon dioxide gas. The pigs were pricked and scalded on the slaughter line.

### Taking pictures for claw scoring

Claw scoring was performed by one trained veterinary via photographs. The claws of all animals were evaluated at suckling piglet age, at 4 weeks of age, and as carcasses to assess the claw asymmetries.

**Fig. 3:**
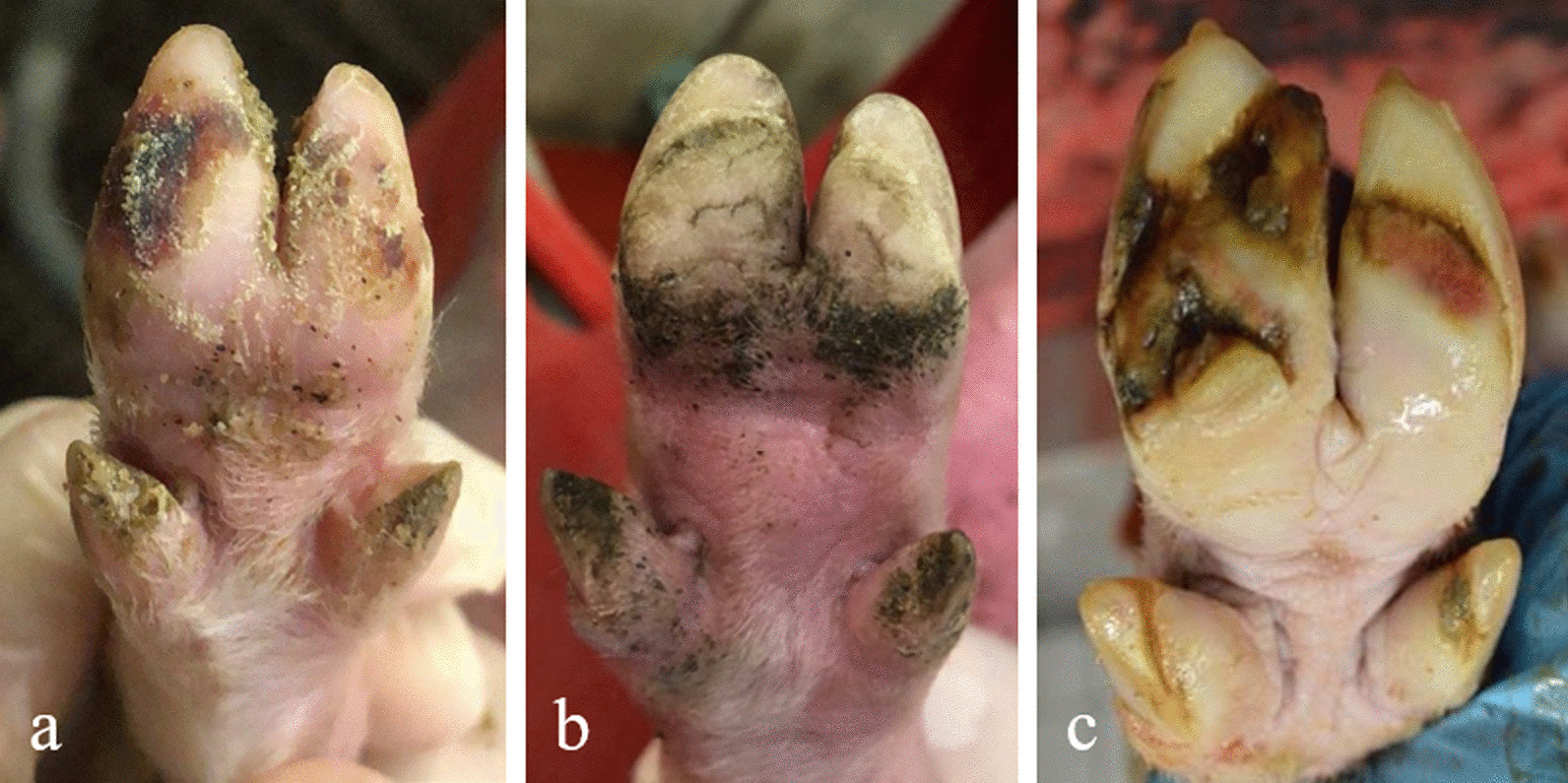
a-c Example photographs showing claw asymmetries at the three observation times. **a** Photograph from a suckling piglet at the first days of life (right hind claw), **b** from a weaned piglet at the age of 4 weeks (right hind claw), and **c** from a final fattened pig at slaughter (right front claw) (pictures taken by Sarah Seufert)

For detailed claw scoring, pictures were taken of all four claws of 515 suckling pigs between their 3rd and 5th day of life for the first time. Two piglets were not included in this first evaluation because of cross-fostering; they joined the study later. Animals were identified via ear tag and the sex was recorded. To take pictures of the claws, the piglets were kept lying on their backs by an assistant, and all four claws were photographed using the back camera of an iPhone 8 (Apple, Cupertino, CA, USA) (Fig. 3a). Care was taken to ensure that the camera was held exactly parallel to the claws to avoid tilted images. The front limbs of the piglets were stretched by the assistant applying light pressure on the elbow joints. Consequently, the angles of the spread claws were almost uniform. The claws of the hind legs were spread automatically due to the supine position.

The weaned piglets’ claws were photographed for the second time at 4 weeks of age and thus shortly before their weaning. Here, 482 of the initial 515 examined animals were still part of the study. The remaining 35 piglets died or were culled. Therefore, the suckling piglet mortality in the first 4 weeks of life averaged 6.8% across the five batches. As mentioned before, cross-fostering two more piglets of the same genetics was part of this second evaluation. Therefore, 482 animals were examined and photographed at weaning age (Fig. 3b). According to the procedure for suckling pigs, the claws were photographed and the ear tag for identification was recorded.

At the time of slaughter, 307 animals remained in the study. The other pigs had died, had been culled, had been slaughtered earlier, or had been sorted out and sold due to low weight. At the slaughter house, pictures of the pigs’ claws were taken at the slaughter line after pricking and before scalding (Fig. 3c). The claws of the front limbs were sprayed with water by the butcher before taking photos to clean them of blood and to make the lesions visible. For hygiene protection, the slaughterhouses’ own camera (Nikon D3200, Nikon Europe BV, Amsterdam, The Netherlands) was used. An ear tag with individual animal identification numbers was recorded, and photos of both front claws as well as of the one unfixed hind leg were taken. Technical limitations implies that only one hind claw of each slaughtered animal was photographed. The distribution was 118 claws of the left hind limb and 189 claws of the right hind limb.

### Scoring claw asymmetries on the photographs

The classification of the claw asymmetries was performed using the photographs to facilitate accurate scoring while minimizing the animals’ stress. A four-level scoring system was used for classification with A-Score 0 representing no asymmetry, A-Score 1 showing claws with slight asymmetries, A-Score 2 with intermediate asymmetry, and A-Score 3 with strong asymmetry (Table 5). The footing areas of the two main claws were compared to determine the degree of asymmetry of the claws. For instance, A-Score 1 was defined as a slight asymmetry with the area of the main claws differing between more than 5–15%. To specify this, the footing areas of the two main claws were measured and compared on 50 representative photographs using IC Measure (version 2.0.0.286, The Imaging Source Europe GmbH, Bremen, Germany).

**Table 5:**
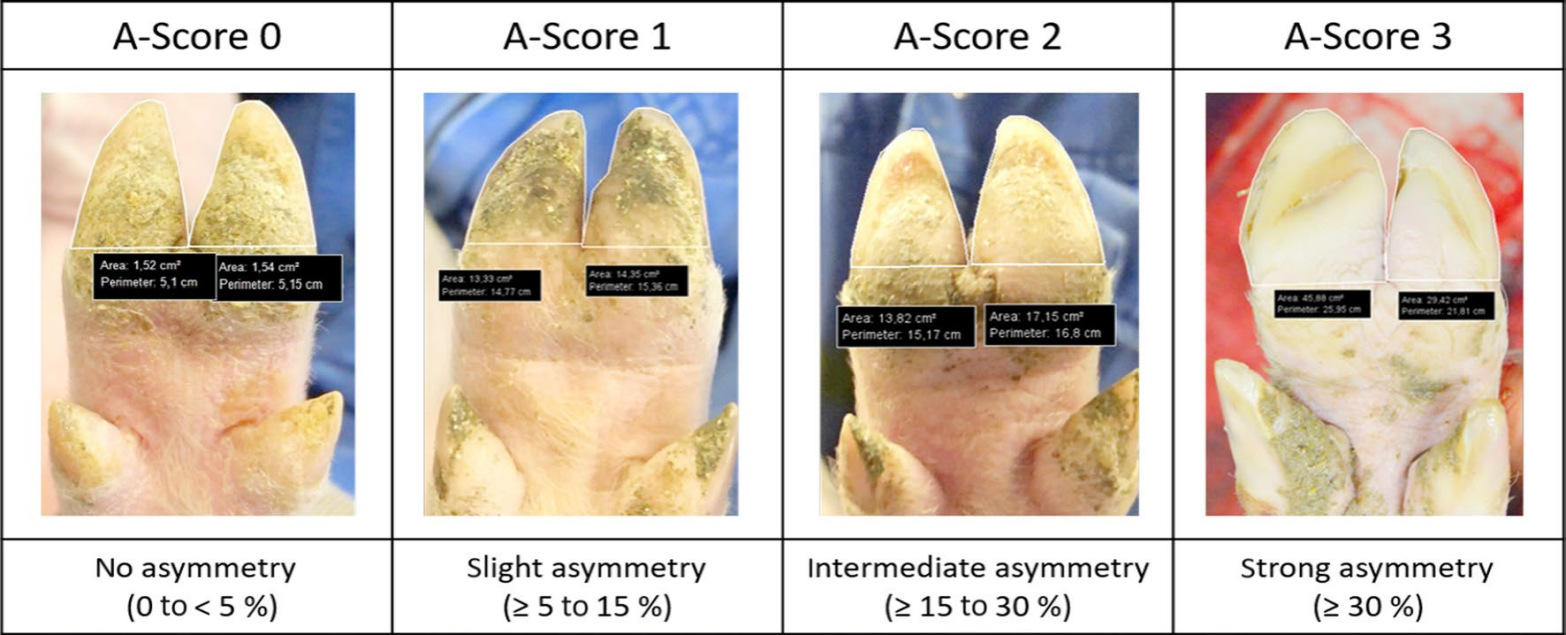
Photographs showing examples for the used scoring system for claw asymmetries (A-Score)

Determination of the area difference between the two main claws on the photographs as basis for the scoring system of claws’ asymmetry. Percentages (%) referring to differences in footing area. A-Score 0 represented by a left hind claw, 4 weeks old; A-Score 1 represented by a left front claw, 4 weeks old; A-Score 2 left hind claw, 4 weeks old; A-Score 3 slaughter pig left front claw (pictures taken by Sarah Seufert)

To ensure observer reliability, 100 randomly selected photographs were scored beforehand by two observers (veterinarian, researcher) using the four-level scoring system. Thus, interobserver agreement was calculated with Krippendorffs’ alpha health [44] and evaluated using the classification proposed by Landis and Koch [45] (< 0.00 = poor; 0.00–0.20 = slight; 0.21–0.40 = fair; 0.41–0.60 = moderate; 0.61–0.80 = substantial; 0.81–1.00 = very good). Afterwards, all photos (n = 4909) were visually classified by one main observer (veterinarian).

### Classification of claw sole ulcerations on the photographs

The occurrence of sole ulcerations was evaluated using claw photographs. The severity of the sole ulceration was classified using a four-level scoring with U-Score 0 showing no visible ulceration, U-Score 1 representing a slight ulceration, U-Score 2 showing an intermediate ulceration, and U-Score 3 representing a strong/severe ulceration (Table 6). Again, 100 randomly selected photographs were scored beforehand by two observers (veterinarian, researcher) using the four-level scoring system. Krippendorffs’ alpha [44] was calculated to ensure observer reliability, and the result was evaluated using the classification proposed by Landis and Koch [45]. Afterwards, all photos (n = 4909 were visually classified by one main observer (veterinarian).

**Table 6:**
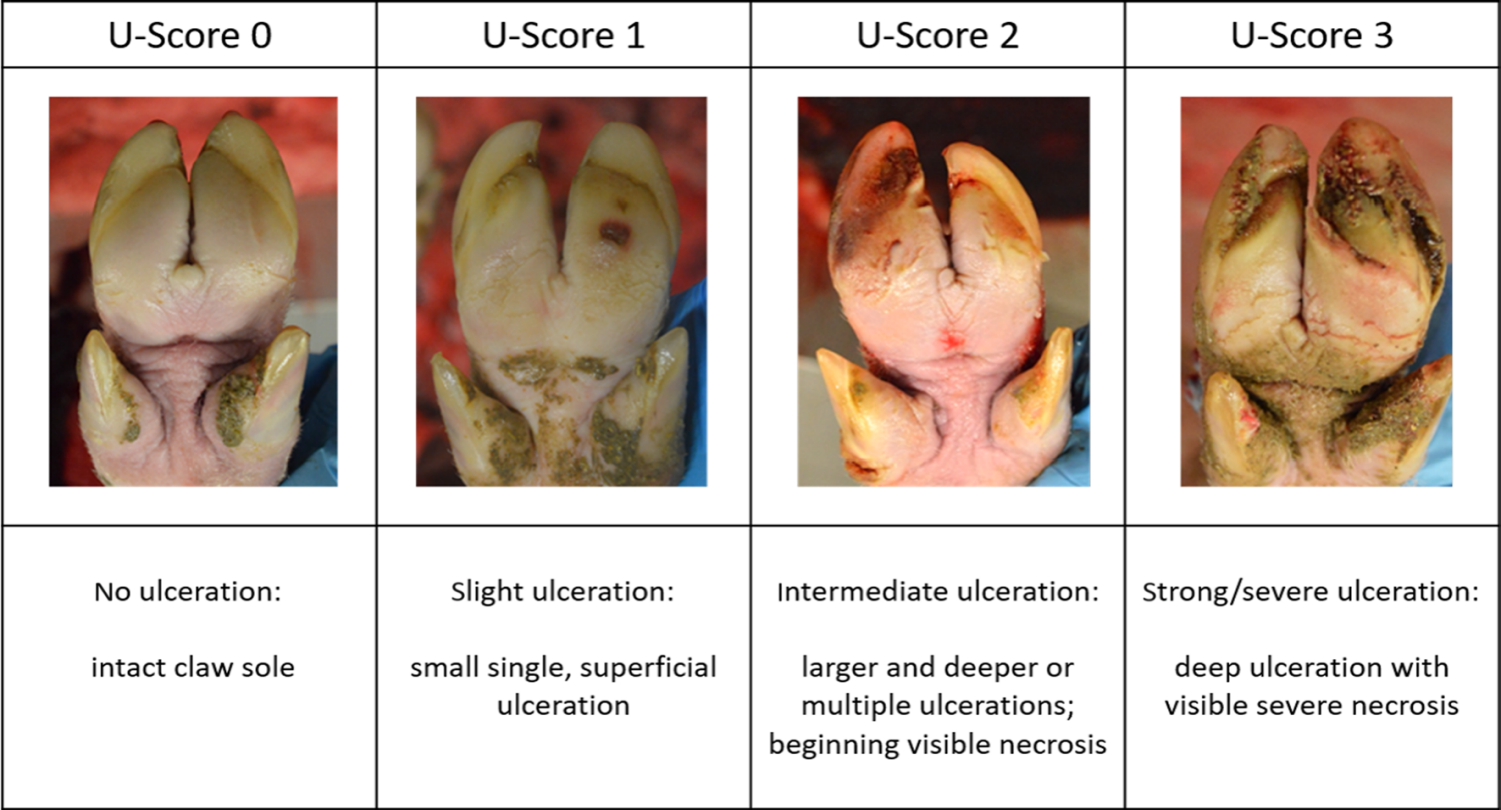
Photographs showing examples of the scoring system for sole ulcerations (U-Score)

U-Score 0: slaughter pig, left front claw; U-Score 1: slaughter pig, left front claw; U-Score 2: slaughter pig, right front claw; U-Score 3: slaughter pig, left front claw (pictures taken by Sarah Seufert)

The next step was to relate the occurrence of claw asymmetries to those of claw sole ulcerations; thus, sole ulceration scores were merged into a two-stage system as follows:

*U-Class 0* representing claws with no visible or slight ulcerations (U-Score 0 and 1).

*U-Class 1* representing claws with intermediate or strong ulceration (U-Score 2 and 3).

### Statistical analysis

All results were processed with Microsoft Excel (Microsoft Corporation, Redmond, WA, USA) and statistically analyzed with SAS version 9.4 (SAS Institute Inc., Cary, NC, USA). The frequency distributions of the occurrence of claw asymmetries and sole ulcerations during lifetime were analyzed on a descriptive basis. To verify the interobserver reliability between the scorings of the two observers, Krippendorffs’ alpha (KALPHA) [44] was applied using the ‘macro’ developed by Hayes [46]. For the occurrence of intermediate to severe sole ulcerations (U-Class 1), the odds ratios (OR) for the maximum rated value of claw asymmetry were calculated using the LOGISTIC procedure. *P*-values ≤ 0.05 were interpreted as statistically significant.

## Data Availability

The datasets used and/or analyzed during the current study are available from the corresponding author on reasonable request.
